# Comparison of Telehealth and Onsite Supervised Maintenance Exercise Programs for Adults With Chronic Lung Disease: Protocol for a Pilot Randomized Feasibility Trial

**DOI:** 10.2196/71039

**Published:** 2025-08-25

**Authors:** Rania Karim, Lesley Smith, Jane Baldwin, Rebecca Pham, Hyun Jung Kim, Lauren Miccile, Michael Sullivan, Shweta Gore

**Affiliations:** 1 Department of Physical Therapy School of Health and Rehabilitation Sciences MGH Institute of Health Professions Boston, MA United States; 2 Department of Physical Therapy Rehabilitation Brigham and Women's Hospital Boston, MA United States; 3 Department of Physical Therapy Massachusetts General Hospital Boston, MA United States

**Keywords:** chronic lung disease, pulmonary rehabilitation, telerehabilitation, exercise capacity, quality of life, health care utilization

## Abstract

**Background:**

Chronic lung diseases (CLDs) such as chronic obstructive pulmonary disease, interstitial lung disease, and chronic asthma contribute significantly to the global burden of disease. Pulmonary rehabilitation (PR) is an effective intervention for improving the exercise capacity, increasing the quality of life, and reducing hospitalizations in individuals with CLD. However, benefits from PR quickly decline following discharge from traditional programs. Telerehabilitation (tele-rehab) has emerged as a potential solution for extending the benefits of PR; yet, few studies have compared its efficacy with conventional onsite maintenance rehabilitation.

**Objective:**

This pilot protocol aims to assess the effectiveness of an 8-week supervised maintenance rehabilitation program delivered onsite or remotely via tele-rehab in patients with CLD.

**Methods:**

This randomized, assessor-blinded feasibility pilot trial will enroll 30 participants who have completed outpatient PR. Participants will be randomized into 2 groups: (1) tele-rehab, involving remotely supervised sessions conducted via videoconferencing or (2) onsite maintenance rehabilitation with in-person sessions. Both groups will receive weekly supervised sessions for 8 weeks facilitated by licensed physical therapists. Assessments will be conducted onsite at baseline, after the 8-week intervention, and at 1-month follow-up by a blinded assessor. The feasibility of the intervention and the study methods will be assessed by the log-in into the number of sessions attended after randomization, the ease of using the equipment and procedures, optimal session length, and adherence to the intervention protocol. Clinical outcomes will include dyspnea and perceived exertion, respiratory muscle strength, physical function, 6-minute walk distance, physical activity, and quality of life. Descriptive statistics with means and standard deviations for continuous and frequency distributions for categorical data will be utilized to report individual and group characteristics. Feasibility outcomes will be compared using independent 2-sided *t* tests. Between-group comparisons will use mixed analysis of variance, and within-group comparisons will be analyzed with a repeated measures analysis of variance.

**Results:**

Recruitment for this institutional review board—approved study (approval date: July 25, 2023) began in January 2024, with 11 (37%) of the 30 participants enrolled to date. This study will generate data comparing tele-rehab and onsite rehabilitation in terms of the primary and secondary outcomes. Use data such as program adherence and attrition will also be reported. The results will inform the feasibility, adherence, and effectiveness of tele-rehab as a sustainable alternative to onsite maintenance rehabilitation. The study is anticipated to be completed by April 2026.

**Conclusions:**

This study hypothesizes that both tele-rehab and onsite programs will significantly improve clinical outcomes, with tele-rehab yielding comparable benefits to onsite maintenance. Findings will provide evidence supporting accessible and sustainable maintenance rehabilitation options for patients with CLD and guide future large-scale efficacy trials.

**Trial Registration:**

ClinicalTrials.gov NCT06304207; https://clinicaltrials.gov/study/NCT06304207

**International Registered Report Identifier (IRRID):**

DERR1-10.2196/71039

## Introduction

Chronic lung disease (CLD), an umbrella term for persistent lung conditions, including chronic obstructive pulmonary disease (COPD), interstitial lung disease, bronchiectasis, and chronic asthma, is a major contributor to the global burden of disease [1,2]. Patients with CLD are often trapped in the downward spiral of mobility, where symptoms such as dyspnea and fatigue limit physical activity [3,4]. Reduced activity levels further accelerate disease progression, leading to declines in muscle strength, deconditioning, and worsening of symptoms [3,4]. Consequently, patients experience increased respiratory exacerbations, resulting in frequent hospitalizations and readmissions, impacting the quality of life and health care costs [5-9].

Pulmonary rehabilitation (PR) has been reported as one of the most evidence-based, cost-effective, and efficient interventions to improve health outcomes in CLD [5-9]. The American Thoracic Society/European Respiratory Society statement defines PR as “a comprehensive intervention based on a thorough patient assessment followed by patient-tailored therapies, which include, but are not limited to, exercise training, education, and behavior change, designed to improve the physical and emotional condition of people with chronic respiratory disease and to promote the long-term adherence of health-enhancing behaviors” [5]. Compared to medical management alone, strong evidence supports PR to improve lung function, exercise capacity, physical function, self-efficacy, quality of life, and physical activity while also delaying disease progression and reducing the risk of hospitalization and mortality [5-9].

Despite strong evidence demonstrating PR effectiveness, reported benefits rapidly decline after discharge in the absence of supervised maintenance as adherence to health-enhancing behaviors and physical activity levels wane over time [10,11]. To promote long-term adherence, patients may need a continuum of care with tapered supervision beyond formal PR. Supervised maintenance programs involve ongoing supervised exercise at a lower frequency than PR programs. Although an abundance of literature supports the benefits of PR, studies examining maintenance programs are scarce and demonstrate mixed results. Limited studies using community-based supervised maintenance following PR have demonstrated a strong potential of these programs in maintaining a variety of outcomes, including physical activity level, quality of life, 6-minute walk distance, and dyspnea scores [12], but other studies on maintenance have failed to demonstrate these benefits [13,14]. Overall, the certainty of evidence on the benefits of maintenance programs is deemed low, particularly due to inconsistent performance on outcome measures, high risk of bias, lack of rigorous methodologies, attrition, and small sample sizes [15]. Ongoing improvements in outcomes are believed to result from enhanced function, exercise tolerance, and symptom control with structured maintenance programs [6,7,15]. However, access barriers and adherence challenges persist, highlighting the need for feasible, scalable models like telehealth [16]. These limitations underscore the need for well-designed studies to evaluate the feasibility and effectiveness of supervised maintenance programs, particularly those delivered via telehealth.

One method to improve adherence in maintenance programs is telerehabilitation (tele-rehab), which is a way of participating in medical appointments via a technology device [17]. Tele-rehab is a viable alternative rehabilitation approach for patients with CLD and holds potential as an effective tool for increasing positive behavioral changes, including long-term adherence and a more physically active lifestyle [18-21]. Few studies [18,22-24] have compared the benefits of tele-rehab versus onsite PR on health outcomes in people living with CLD, and even fewer studies have compared supervised maintenance programs delivered via tele-rehab with onsite maintenance, leaving many gaps in the literature [25]. Furthermore, most studies are limited to people with COPD, with only a small number investigating the effects of tele-rehab on other CLDs [26,27].

Given these limitations, there is a clear need for well-designed feasibility studies to evaluate the effectiveness and practicality of supervised maintenance programs, particularly those delivered via telehealth. The purpose of this pilot feasibility trial is to (1) assess the feasibility of delivering an 8-week supervised maintenance program remotely via tele-rehab in comparison to onsite delivery and (2) compare the effectiveness of these interventions on clinical outcomes in patients with CLD. We hypothesize that tele-rehab will be feasible to implement, with acceptable adherence, and will yield comparable improvements in clinical outcomes to onsite rehabilitation.

## Methods

### Ethical Considerations

The study protocol has been reviewed and approved by the institutional review board at Massachusetts Brigham (approval 2023P000708). The study protocol is registered on Clinicaltrials.gov (NCT06304207). Any changes to the protocol will be reported to the institutional review board by the principal investigator. Participants will provide verbal consent during a phone call after being prescreened for eligibility. A signed consent form will then be obtained either in person during their baseline assessment or electronically through Research Electronic Data Capture (REDCap) [28]. Institutional affiliations (eg, Massachusetts Brigham and the MGH Institute of Health Professions) will be transparently presented to the participants during recruitment to ensure trust and informed decision-making. In order to protect the participant's privacy, all recorded data will be deidentified and stored in a password-protected computer. This information will then be destroyed at the end of the study. All study team members underwent training on protecting data privacy. To reduce the barriers to participation and to encourage enrollment, we will provide free parking for onsite visits; an iPad will be loaned for the study duration if needed for tele-rehab, and a US $50 gift card will be provided upon study completion.

### Study Design

**Figure 1:**
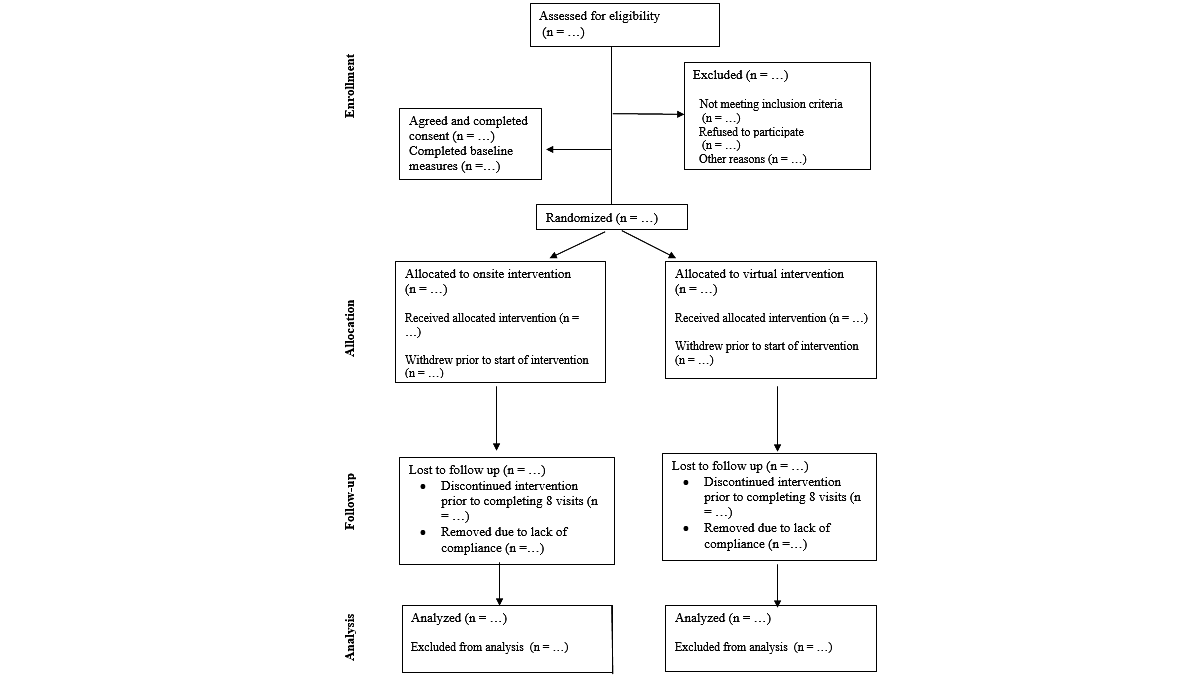
Participant Recruitment and Allocation.

This study follows the CONSORT (Consolidated Standards of Reporting Trials; Multimedia Appendix 1) and SPIRIT (Standard Protocol Items: Recommendations for Interventional Trials; Multimedia Appendix 2) guidelines. The study will be a randomized, assessor-blinded, pilot feasibility trial. Participants will be randomly assigned in a 1:1 allocation ratio to either (1) a tele-rehab group with remotely supervised intervention or (2) an onsite group with in-person supervision. Figure 1 outlines the study design and recruitment process. The anticipated study period is from September 1, 2023, to April 1, 2026.

Given the higher prevalence of CLD in late adulthood [29-31], individuals aged 40 years and older with a physician-confirmed diagnosis of CLD will be recruited for this study. Inclusion criteria include the ability to understand English instructions, walk independently with or without the use of mobility devices, complete a 6-minute walk test (6MWT) while maintaining oxygen saturation of at least 85% with or without supplemental oxygen, a history of onsite rehabilitation completion, and internet access, which will be verified by accessing email for communication. Exclusion criteria include medical conditions causing significant mobility limitations such as stroke, Parkinson disease, multiple sclerosis, or severe degenerative joint disease. Participants will also be excluded if they are unable to maintain oxygen saturation of at least 85% during 6MWT, have baseline hemodynamic instability (eg, unstable angina, recent myocardial infarction within 3 months, uncontrolled atrial fibrillation), advanced heart failure (New York Heart Association Class IV), or require mechanical circulatory support such as ventricular assist devices.

### Participant Enrollment

Eligible participants will be recruited through clinical settings, offline outreach, and direct contact by study team members. During recruitment, participants will receive detailed verbal information about the study, including the randomization process, assessments, and intervention details.

### Study Procedures

#### Onsite Initial Evaluation

After providing informed consent, all participants will complete a baseline assessment at Dr Charles and Ann Saunders IMPACT Practice Center at MGH Institute of Health Professions. This initial visit, conducted by an assessor blinded to group allocation, will serve as the index date, referred to as time point 0 (time0). Since recruitment will occur on a rolling basis, time0 will vary for each participant. During the baseline visit, the assessor will review the self-monitoring of vitals and symptom provocation with participants. Education on monitoring oxygen saturation by using pulse oximetry, rate of perceived exertion, and heart rate will be provided in detail. Additionally, participants will receive a pulse oximeter and a Threshold Inspiratory Muscle Trainer Respironics positive expiratory pressure device during the baseline visit to use throughout the study.

#### Random Assignment

Following the baseline assessment, a separate research team member will contact each participant via phone to communicate the randomized group assignment. Participants will be randomly assigned to one of the 2 groups: (1) tele-rehab, where the participants receive supervised intervention remotely at home or (2) onsite outpatient rehabilitation. A web-based randomization service [32] will be used to generate random assignments to eliminate allocation bias.

#### Outcome Assessments

Outcomes will be recorded in-person at 3 time points: at baseline before the intervention (time0), at the end of the 8-week intervention (time1), and at 1-month post intervention during follow-up (time2). A blinded study team member will conduct all outcome assessments at each time point (Table 1), with reminder emails sent to participants during the week the assessment is scheduled. Feasibility outcomes will include the number of sessions attended post randomization, the ease of using equipment and procedures, optimal session length, and adherence as measured by the daily activity log. Clinical outcomes will include baseline vitals such as heart rate, respiratory rate, oxygen saturation measured with a pulse oximeter, Borg rating of perceived exertion [33], and modified Medical Research Council dyspnea scale [34,35]. Respiratory muscle strength will be evaluated using maximal inspiratory pressure, while physical function will be assessed with the 30-second chair rise test [36] and the 10-meter walk test [37]. Functional capacity will be measured through 6MWT [38,39] and the 10-meter incremental shuttle walk test [40]. Physical activity will be assessed using the International Physical Activity Questionnaire [41], and the quality of life will be evaluated with the Patient-Reported Outcomes Measurement Information System Global Health Questionnaire [42] and the COPD Assessment Test [43]. Secondary outcomes will include planned or unplanned physician visits, emergency room visits, falls, and injuries.

**Table 1:** Interventions provided for tele-rehab^a^ and onsite rehab^b^ groups.

Goal (duration)	Tele-rehab group and onsite rehab group
Check-in and education (5 min)	Review of activities during the week at home Symptom changes during the week Education on self-management of airway clearance Education on the importance of maintaining regular physical activity
Breathing retraining (5 min)	Inspiratory muscle training using a Threshold Inspiratory Muscle Trainer at 30%-50% of maximal inspiratory pressure
Warm-up (5 min)	Spot marching Stretching large group muscles
Aerobic exercise (25 min)	Treadmill/cycle ergometer/NuStep/walking/stair climbing^c^ Treadmill walking: initial walking speed at 60%-85% of peak VO2^d^ estimated from 6MWT^e^ and/or rate of perceived exertion of 13-16 (somewhat hard to hard) corresponding to 60%-85% of peak VO2 Cycle ergometer and NuStep intensity at rate of perceived exertion of 13-16 or a resistance corresponding to 60%-85% of peak VO2 derived from published equations Overground walking or stairs at rate of perceived exertion of 13-16 (somewhat hard to hard) corresponding to 60%-85% of peak VO2
Functional strengthening (15 min)	Partial squats, sit-to-stand exercise, lunges, and step-ups/downs 3 sets of 10 repetitions each
Cool down (5 min)	Spot marching Stretching of large group muscles

^a^Tele-rehab: telerehabilitation group. This group receives remotely supervised sessions conducted via videoconferencing.

^b^Onsite-rehab: onsite maintenance rehabilitation. This group receives onsite maintenance rehabilitation with in-person sessions.

^c^No stair climbing in the onsite rehabilitation group.

^d^VO_2_: oxygen consumption.

^e^6MWT: 6-minute walk test.

#### Intervention

Participants will begin the 8-week intervention for the PR maintenance program in either the onsite or remote group depending on their group allocation, with supervised visits occurring once a week for both groups and regular reminders for the meetings sent to participants. At the initial treatment session, each participant will receive printed educational materials from the COPD Foundation [44] and the Pulmonary Fibrosis Foundation [45]. These resources will cover topics such as living well with CLD, the impact of smoking, and creating a CLD action plan, including disaster preparedness. Both groups will participate in a weekly 60-minute supervised session. Supervised sessions will be delivered in-person for the onsite group and remotely via video conferencing for the tele-rehab group. The intervention intensity will be determined by the distance covered in 6MWT conducted at baseline. Licensed physical therapists from the study team who have expertise in managing chronic conditions will deliver all the interventions. These therapists will receive specific training on the study protocol to ensure consistency in intervention delivery. All interventions will adhere to the research protocol outlined in Table 1.

#### Onsite Group

The supervised 60-minute session for onsite participants will include multiple components. The session will begin with a brief check-in to review the participant's weekly activities at home and any changes in symptoms, followed by education on self-management strategies for airway clearance and the importance of maintaining regular physical activity. Breathing retraining will involve inspiratory muscle training using the Threshold Inspiratory Muscle Trainer Respironics PEP at 30% to 50% of maximal inspiratory pressure. After the breathing exercises, participants will complete a 25-minute aerobic exercise training session, preceded by a short warm-up. Aerobic training will be performed using available equipment such as a NuStep, treadmill, cycle ergometer, or through overground activities, including walking or stair climbing. The training intensity will be prescribed based on the baseline 6MWT results. Peak oxygen consumption (VO_2_) will be estimated using published regression equations [46]. Training will be conducted at 60%-85% of VO_2_ reserve and converted to walking distance and speed using the American College of Sports Medicine walking equations for treadmill and overground walking [47]. For nonwalking activities such as cycling or stair climbing, a corresponding rate of perceived exertion of 13-16 will be used. Aerobic training may be completed as a continuous 25-minute session or divided into intervals (eg, 2 × 12.5 minutes or 3 × 8.3 minutes) if symptoms limit continuous exercise.

Following aerobic training, functional strength exercises focusing on lower extremity muscles, including partial squats, sit-to-stand exercises, lunges, and step-ups/step-downs, will be performed. Each session will conclude with a short cool-down period. The details of the intervention sessions are outlined in Table 1. Participants will also be asked to maintain a daily physical activity log and report any planned or unplanned physician visits, emergency room visits, falls, or injuries. The attendance for each session will be recorded.

#### Tele-Rehab Group

Participants in the tele-rehab group will follow the same intervention protocol as the onsite group described above, with the mode of aerobic exercise adjusted based on available equipment (see Table 1). Each tele-rehab participant will receive a respiratory muscle training inspiratory muscle trainer device for home use. The intervention will be delivered remotely via established tele-rehabilitation models using a tablet computer. iPads will be loaned to participants as needed for the duration of the study. Videoconferencing will be conducted via research-grade Zoom teleconferencing [48], which will enable participants to see and speak to the supervising physical therapist. To ensure the safety of participants and proper understanding of equipment use and the exercise program, we will conduct the initial training session and home exercise program setup during the initial onsite visit. The fidelity of the exercise intervention for both groups will be maintained through regular staff training, audits of exercise prescription and progression, and monitoring of participant engagement.

### Statistical Analysis

Since a large 2-group comparative study has not yet been published, this pilot study will generate an estimate of variance and the percentage of change in outcomes needed to determine the power and sample size for a larger study. Therefore, estimation rather than statistical testing is the main objective of this study. Given the possibility of attrition from dropouts, we will recruit 40 participants with a goal to have 30 participants complete the study (15 in each group). Descriptive statistics will be used to summarize the demographic and clinical characteristics and outcomes for all the participants. Feasibility will be assessed using frequency distributions of session adherence and use of activity logs. Comparisons between groups on all outcomes at preintervention and postintervention will be conducted with the intention to treat using a mixed analysis of variance. Within-group comparisons at all 3 time points will be conducted via a repeated measures analysis of variance. Adherence to the program between groups will be compared using independent 2-sided *t* tests. Finally, descriptive statistics with frequency distributions of adverse events, planned and unplanned physician visits, and frequency of use of daily activity log will be reported.

## Results

This study received institutional review board approval from Massachusetts Brigham (approval 2023P000708) on July 25, 2023.The recruitment for the first wave began in January 2024. To date, we have enrolled 11 (37%) out of 30 participants, as projected. The final wave of recruitment will end by December 2025, and the final participant will complete the study by March 30, 2026. Descriptive data revealed that 1 participant in the virtual group who completed the study had better 6MWT scores than the participant in the onsite group. We will start analyzing the complete responses by April 30, 2026, and the publication of results is expected at the end of 2026.

## Discussion

### Overview

We describe a protocol for a pilot randomized feasibility clinical trial to examine the effectiveness of virtually delivered supervised maintenance for patients with CLD. This proposed intervention breaks down barriers to equitably ensure sustained benefit and improved self-efficacy for all, regardless of their ability to physically reach an onsite location. Despite level 1 evidence from high-quality clinical trials supporting the effectiveness of PR in improving health outcomes, barriers to accessing physical locations have historically limited the utilization of these services. More importantly, even for those who complete the onsite rehabilitation, the benefits from rehabilitation do not last following discharge. As finding ways to improve self-efficacy and long-term adherence are key to preventing adverse outcomes, examining maintenance programs is crucial. This pilot trial will provide information on the feasibility of the intervention in a remote setting and will pave the way for large-scale clinical trials.

### Limitations

There are several limitations that we would like to acknowledge. Although we anticipate achieving a feasible, acceptable, and high-fidelity protocol, it is possible that some challenges remain. Given a feasibility pilot, we anticipate being underpowered to assess intervention efficacy. Despite the randomized design, allocation concealment is not possible. The risk of participants dropping out after learning their allocation assignment cannot be ruled out. We have taken measures to minimize this risk by having trained research staff instead of staff manage action planning and SMS text messaging procedures to avoid any accidental information exchange about other participants. Although steps are taken to improve access to all potential individuals, limiting inclusion to participants who speak English may limit the reach to participants who have limited English proficiency.

### Conclusions

This pilot randomized feasibility trial aims to address critical gaps in the long-term management of CLD by comparing onsite and tele-rehab supervised maintenance programs. The study emphasizes the importance of overcoming traditional barriers to PR, such as transportation challenges and limited facility availability while promoting equitable access to care. By focusing on improving self-efficacy and adherence to health-enhancing behaviors, this intervention has the potential to sustain the benefits of PR beyond discharge and reduce adverse health outcomes. The trial will provide valuable data on feasibility, adherence, and clinical outcomes for tele-rehab and onsite programs, providing insights for the development of larger-scale trials. Additionally, this study addresses the growing need for innovative care models that incorporate technology to enhance patient outcomes and reduce health care disparities. Despite the acknowledged limitations such as the small sample size, language restrictions, and the potential for dropout, the findings will contribute valuable information to guide future efficacy trials. This trial has the potential to inform future standards of care for patients with CLD by demonstrating the feasibility of accessible and effective tele-rehabilitation maintenance programs to sustain long-term health benefits.

## Appendix

Multimedia Appendix 1 CONSORT-eHEALTH checklist (V 1.6.1).

Multimedia Appendix 2 SPIRIT checklist for reporting the protocol.

## Abbreviations

6MWT: 6-minute walk test
CLD: chronic lung disease
CONSORT: Consolidated Standards of Reporting Trials
COPD: chronic obstructive pulmonary disease
PR: pulmonary rehabilitation
REDCap: Research Electronic Data Capture
SPIRIT: Standard Protocol Items: Recommendations for Interventional Trials
Tele-rehab: telerehabilitation
VO2: oxygen consumption

## Data Availability

The datasets generated or analyzed during this study will be available from the corresponding author on reasonable request.
